# Neural correlates of victimization in psychosis: differences in brain response to angry faces

**DOI:** 10.1038/s41537-019-0082-z

**Published:** 2019-09-09

**Authors:** Elisabeth C. D. van der Stouwe, Jooske T. van Busschbach, Esther M. Opmeer, Bertine de Vries, Jan-Bernard C. Marsman, André Aleman, Gerdina H. M. Pijnenborg

**Affiliations:** 1 University of Groningen, University Medical Center Groningen, University Center of Psychiatry, Rob Giel Onderzoekcentrum, Hanzeplein 1, 9713 GZ Groningen, the Netherlands; 2 Department of Neuroscience, Cognitive Neuroscience Center, University of Groningen, University Medical Center Groningen, Antonius Deusinglaan 2, 9713 AW Groningen, the Netherlands; 3 Department of Movement and Education, Windesheim University of Applied Sciences, Campus 2-6, 8017 CA Zwolle, the Netherlands; 4 Department of Clinical Psychology, University of Groningen, Grote Kruisstraat 2/1, 9712 TS Groningen, the Netherlands; 5 Department of Psychotic Disorders, GGZ-Drenthe, Dennenweg 9, 9404 LA Assen, the Netherlands

**Keywords:** Limbic system, Psychosis

## Abstract

Individuals with psychosis are at an increased risk of victimization. Processing of facial expressions has been suggested to be associated with victimization in this patient group. Especially processing of angry expressions may be relevant in the context of victimization. Therefore, differences in brain activation and connectivity between victimized and nonvictimized patients during processing of angry faces were investigated. Thirty-nine patients, of whom nineteen had experienced threats, assaults, or sexual violence in the past 5 years, underwent fMRI scanning, during which they viewed angry and neutral facial expressions. Using general linear model (GLM) analyses, generalized psychophysiological (gPPI) analysis and independent component analyses (ICA) differences in brain activation and connectivity between groups in response to angry faces were investigated. Whereas differences in regional brain activation GLM and gPPI analyses yielded no differences between groups, ICA revealed more deactivation of the sensorimotor network in victimized participants. Deactivation of the sensorimotor network in response to angry faces in victimized patients, might indicate a freeze reaction to threatening stimuli, previously observed in traumatized individuals.

## Introduction

Individuals with a diagnosis in the psychotic spectrum are at an increased risk of becoming the victim of a crime.^1^ According to a meta-analysis on victimization in this group, the median prevalence rate for victimization during adulthood is 66% for violent victimization (e.g., physical assault, threat with violence or with a weapon), 27% for sexual victimization (e.g., forced sexual penetration, sexual touch without consent, or sexual harassment) and 39% for nonviolent crime (e.g., theft of property or money and fraud).^2^ One of the processes associated with victimization in people with a psychotic disorder may be social cognition,^3^ particularly problems in processing facial expressions.^4^

Individuals with a psychotic disorder have consistently shown inadequate processing of facial expressions.^5,6^ Especially processing of angry facial expressions might be relevant in the context of personal victimization (e.g., violent threats and assaults), because adequate processing of angry faces enables recognizing the intentions of potential perpetrators. Subsequently, an individual may adequately adjust his/her behavior for example by leaving a social setting, by trying to de-escalate a potential escalation or by showing an assertive reaction. Patients may either perceive faces as less angry and show a decreased response to angry faces^4^ resulting in the absence of an appropriate cue to adjust behavior accordingly, or as more angry and show an increased response to angry faces,^7^ which consecutively may lead to conflicts ultimately resulting in victimization. In turn, victimization is a specific type of trauma which, according to the literature, may elicit either increased sensitivity to threatening stimuli such as angry faces^8^ or induce a rather blunted or freezing response.^9^ In all, although the direction of causality is unclear, the literature suggests an association between alterations in emotional face processing and victimization incidents.^3,4^

To date, research has focused on personal and environmental factors associated with victimization,^2^ with little emphasis on potential related neural processes that could provide important insights in associated mechanisms. Research has revealed a collective network of brain areas to be involved in the processing of facial social information. First of all, the superior temporal sulcus (STS) and the fusiform gyrus (FG) have been implicated in face processing. While the FG responds most strongly to tasks focusing on facial identity, the STS has been found to monitor and interpret behavior of others by responding mostly to changing aspects of faces such as movements of the eyes or mouth.^10–12^ Secondly, the amygdala responds to emotionally and socially relevant information.^13^ Another key area is the insula, which is involved in processing aversive emotions such as disgust, fear, and anger.^14^ The orbitofrontal cortex has been found to monitor future outcomes of social behavior.^15^ Finally, the anterior cingulated cortex (ACC) has projections to both the amygdala and the prefrontal cortex and is therefore, among other functions, implicated in emotion regulation and monitoring of the saliency of emotional information.^16^

A large body of research revealed aberrant neural activation of mentioned areas to be associated with emotional face perception in individuals diagnosed with a psychotic disorder. A recent meta-analysis found that, compared to the general population, people with schizophrenia showed decreased activation throughout the emotional face processing areas (e.g., FG, amygdalala, insula, ACC, medial frontal gyrus, para-hippocampal gyrus, and right medial dorsal thalamus), and increased activation in higher-order visual processing regions within the cuneus.^17^ Although the nature of this increased activation is unclear, it has been suggested that overactivation within higher-order visual regions may compensate deficits in integrating visual information.

Although the current study aimed to investigated neural processes related to victimization in patients with a psychotic disorder, previous studies have investigated processing of threatening stimuli in traumatized individuals. Several studies reported increased brain activation to negative facial expressions, for example in the amygdala, hippocampus, insula, ACC, angular gyrus, supramarginal gyrus, and middle temporal gyrus^18–20^ or increased functional connectivity between the left IFG and the right IFG and right insula during a ToM task.^21^ Other studies reported decreased brain activation of thalamus, the ACC, and the medial frontal gyrus^22^ or decreased resting-state network connectivity within the default mode network, salience network, sensorimotor network, and auditory network during.^23^ In all, studies on trauma mostly revealed differences in brain activation patterns between a traumatized and nontraumatized group of participants.

As for now little is known about the association between emotional face processing and incidences of violence in adults with psychotic disorder. Therefore, the aim of the current study was to investigate whether in patients with a psychotic disorder, those with a history of recent victimization show differences in brain activation and in brain connectivity during processing of angry facial expressions. Based on previous studies with traumatized individuals we hypothesized that in people with a psychotic disorder, victimization is associated with aberrant brain activation and connectivity within brain areas involved in processing of facial expressions.

## Results

### Sample characteristics

Demographical and clinical characteristics are reported in Table 1. The victimized and nonvictimized groups did not differ on sociodemographic or illness related characteristics. Because depression and paranoia may influence processing of facial expressions,^24,25^ we also explored group differences on the individual PANSS depression item and paranoia item, but no differences were found. In the victimized group participants had significantly higher scores on the trauma screening questionnaire. In the nonvictimized group more people used antipsychotic medication and antidepressants.

**Table 1:** Demographic and clinical characteristics

	Victimized participants	Nonvictimized participants	Test statistic
*N*	19	20	
Age, mean (SD)	32 (10.1)	36.2 (11.0)	*t*(37) = −1.24, *p* = 0.22
Gender, *n* (%) male	11 (57.9)	15 (75)	*X*^*2*^(1) = 1.28, *p* = 0.32
*Occupational status, n (%)*			*X*^*2*^(1) = 0.67, *p* = 0.52
Job or voluntary work	7 (36.8)	10 (50.0)	
Unemployed	12 (63.2)	10 (50.0)	
*Living situation, n (%)*			
Alone	8 (42.1)	14 (70)	*X*^*2*^(3) = 3.01, *p* = 0.38
Partner	3 (15.8)	1 (5)	
Family/parents	2 (10.5)	3 (15)	
Supported housing	6 (31.6)	2 (10)	
Age of onset, mean (SD)^a^	19.4 (8.0)	21.5 (9.1)	*t*(34) = −0.74*, p* = 0.47
Number of psychotic episodes, mean (SD) ^a^	4.5 (4.2)	3.6 (3.1)	*t*(34) = 0.73*, p* = 0.47
Number of admissions, mean (SD)^a^	1.4 (1.5)	1.6 (1.2)	*t*(34) = −0.49, *p* = 0.63
*PANSS score, mean (SD)*			
Total	48.4 (9.7)	48.5 (8.3)	*u* *=* 182, *p* = 0.82
Positive	13.1 (3.9)	12.1 (3.5)	*u* *=* 159, *p* = *0.38*
Negative	10.0 (2.5)	10.6 (2.9)	*u* *=* 171, *p* = 0.59
General	25.3 (5.1)	25.9 (4.6)	*u* *=* 181, *p* = 0.79
BNSS total score	15.6 (10.4)	15.4 (9.1)	*u* = 180, *p* = 0.79
PANSS depression item	2.7 (1.6)	2.8 (1.4)	*u* *=* 184, *p* = 0.86
PANSS paranoia item	2.8 (2.5)	2.0 (1.0)	*u* = 152, *p* = 0.26
TSQ, *N* (%) traumatized	5 (26.3)	1 (5)	*p* = 0.18
TSQ, mean (*SD)*symptoms	3.55 (3.25)	1.37 (2.17)	*t*(37) *=* 2.45, *p* = 0.02
*Diagnosis, n (%)*			
Paranoid schizophrenia	3 (15.8%)	8 (40.0%)	
Schizophreniform disorder	2 (10.5%)	2 (10.0%)	
Delusion disorder	3 (15.8%)	0 (0.0%)	
Brief psychotic disorder	1 (5.3%)	1 (5.0%)	
Psychotic disorder NOS	10 (52.6%)	9 (45.0%)	
*Antipsychotic medication, n (%)*			*p* = 0.04
Risperidone	4 (21.1)	4 (20.0)	
Olanzapine	2 (10.5)	4 (20.0)	
Clozapine	1 (5.3)	6 (30.0)	
Aripiprazole	2 (10.5)	6 (30.0)	
Quetiapine	3 (15.8)	2 (10.0)	
Haloperidol	2 (10.5)	1 (5.0)	
Paliperidone	1 (5.3)	1 (5.0)	
Bromperidole	0	1 (5.0)	
Penfluridole	0	2 (10)	
None	6 (31.6)	0	
*Antidepressant medication, n (%)*			*p* = 0.01
Citalopram	5 (25.0)	4 (20.0)	
Venlafaxine	0	3 (15.0)	
Amitriptyline	0	1 (5.0)	
Norpriptyline	0	1 (5.0)	
Lithium	0	1 (5.0)	
Clomipramine	0	1 (5.0)	
Mirtazapine	1 (5.0)	1 (5.0)	
None	14	8	

^a^Data of only 17 victimized participants and 19 nonvictimized participants were available

### Behavioral results

Both groups performed similarly on the gender discrimination task in terms of accuracy (victimized group 74.5% correct [SD 20.7%], nonvictimized group 75.7% correct [SD 16.2%]). There were no differences in performance between groups [*t* = −0.20, *p* = 0.84). There were no differences in RT between conditions (*F*(1) = 3.0, *p* = 0.09) or between groups ((*F*(1) = 1.2, *p* = 0.28). There was also no interaction effect between emotion condition and victimization group (*F*(1) = 0.2, *p* = 0.63). Similarly, there were no differences in accuracies between conditions (*F*(1) = 2,6, *p* = 0.11). There was also no interaction effect between emotion condition and group (*F*(1) = 0.8, *p* = 0.36).

### fMRI results

#### Main effects

During angry faces compared to baseline, the occipital lobe, FG, dorsal anterior, and middle cingulate cortex, superior temporal gyrus, lingual gyrus, calcarine gyrus, middle and inferior frontal gyrus, superior and inferior orbitofrontal gyrus, hippocampus, cuneus, insula, and thalamus were found to be more activated (Table 2). A similar pattern of brain regions were identified for the contrast neutral > baseline (Table 2). Comparing the angry condition with the neutral condition, no differential activation was found (Table 2).

**Table 2:** Peak activations of brain regions, which showed differential activation for the contrasts (angry > baseline) and (neutral > baseline)

Contrast	Regions	Cluster size	*T* value	*Z* value	MNI coordinates		
					*x*	*y*	*z*
Angry > baseline	*Fusiform gyrus*, middle occipital gyrus, inferior occipital gyrus, *superior temporal gyrus*, lingual gyrus, calcarine gyrus, hippocampus, *amygdala*, thalamus, anterior cingulate cortex, middle cingulate cortex	391	17.55	Inf	−39	−76	−16
	*Fusiform gyrus*, middle occipital gyrus, inferior occipital gyrus, *superior temporal gyrus*, lingual gyrus, calcarine gyrus, hippocampus, *amygdala*, thalamus, anterior cingulate cortex, middle cingulate cortex	400	14.19	Inf	36	−76	−16
	*Cuneus*, superior occipital gyrus, middle occipital gyrus	276	11.95	7.65	12	−91	17
	Precentral gyrus, *insula, Superior orbitofrontal gyrus*, inferior orbitofrontal gyrus	507	9.27	6.66	−57	5	17
	Inferior frontal gyrus, precentral gyrus, middle frontal gyrus, *insula, dorsal anterior cingulate cortex*, middle cingulate cortex	666	8.94	6.52	45	8	26
	*Superior temporal gyrus*	154	8.06	6.12	48	−49	11
Neutral > baseline	*Fusiform gyrus*, middle occipital gyrus, inferior occipital gyrus, lingual gyrus, calcarine gyrus, hippocampus, thalamus, anterior cingulate cortex, middle cingulate cortex	319	14.65	Inf	−33	−76	−16
	*Fusiform gyrus*, middle occipital gyrus, superior occipital gyrus, lingual gyrus, calcarine gyrus,	394	14.10	Inf	36	−76	−16
	*Cuneus*, superior occipital gyurs, middle occipital gyrus	284	11.15	7.98	18	−91	14
	Inferior frontal gyrus, precentral gyrus	536	8.70	6.41	42	8	26
	Inferior frontal gyrus, precentral gyrus, *insula, superior orbitofrontal gyrus, anterior cingulate cortex*, middle cingulate cortex, superior motor area	385	8.12	6.15	−48	5	26
	Superior temporal gyrus, middle temporal gyrus	97	7.86	6.02	51	−49	11

**Table 3:** Personal victimization items from the victimization subscale of the Dutch Crime and Victimization Survey

Items	Yes, in the last year	Yes, in the previous year	Yes, in the previous 5 years	No
Did anyone threaten you by means of hitting, kicking, a gun, a knife or something similar in the past 5 years?	5 (12.8%)	1 (2.6%)	4 (10.3%)	29 (74.4%)
Did anyone attack you or abuse you by means of hitting, kicking, a gun, a knife or something similar in the past 5 years?	5 (12.8%)	1 (2.6%)	5 (12.8%)	31 (79.5%)
’Sometimes people touch or grab someone else with sexual intentions in a hurtful manner. Did this happen to you the past years?	2 (5.1%)	2 (5.1%)	5 (12.8%)	34 (87.2%)
Aside from mentioned events, did you become victim of another crime or criminal attempt? If yes, can you describe what happened?	5 (12.8%)	3 (7.7%)	7 (17.9%)	27 (69.2%)

### Group differences in activation

The groups did not differ in brain response to angry > neutral stimuli at a threshold of *p* < 0.05 FWE cluster-level for the extent of the regions of interest (ROI)-mask with an initial threshold of *p* < 0.001 uncorrected.

### Functional connectivity gPPI analysis

Connectivity analysis showed no significant connectivity from the amygdala seed to the rest of the brain. Moreover, no significant differences between the victimized and nonvictimized groups were found in connectivity from the amygdala.

### Independent component analysis

**Fig. 1:**
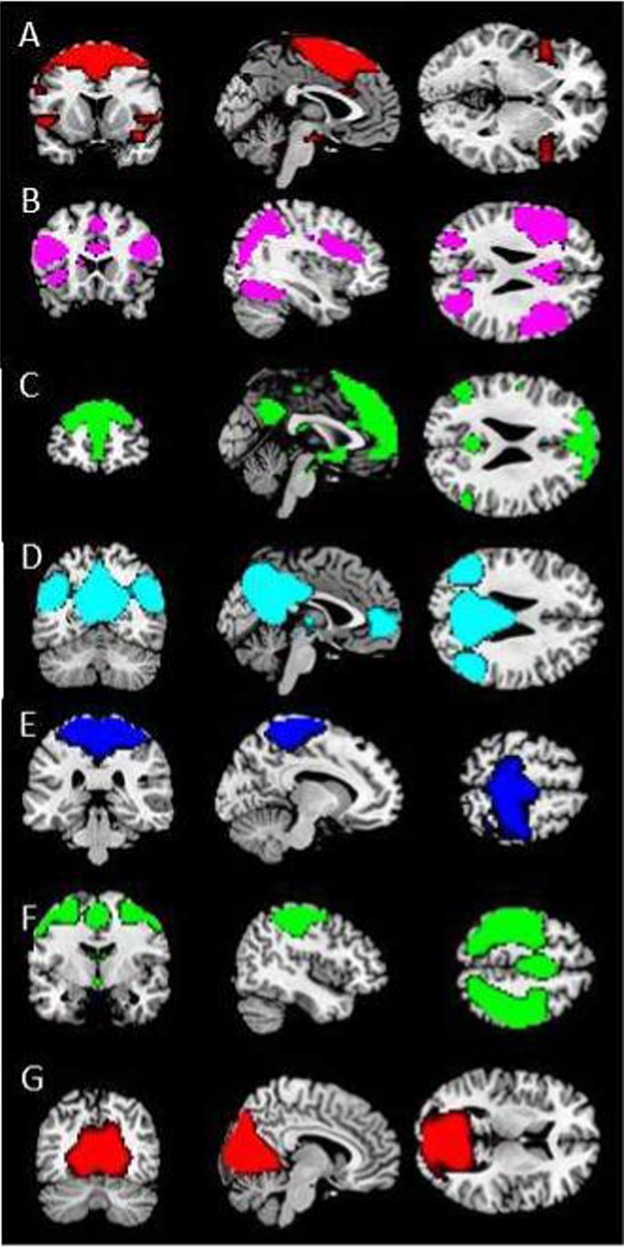
The spatial maps of **a** the salience network (*r* = 0.14), **b** the fronto-parietal network (*r* = 0.13), **c** the anterior default mode network (DMN, *r* = 0.09), **d** the posterior DMN (*r* = 0.10), **e** the medial sensorimotor network (*r* = 0.11), **f** the lateral sensorimotor network (*r* = 0.20), **g** the visual network (*r* = 0.11)

Based on temporal sorting of the 30 components, the eight components that showed the highest correlation with the task were selected. Of these eight components, the first component consisted mostly of cerebrospinal fluid and was therefore excluded. The other seven components resembled components identified in previous resting-state studies^26,27^ and were used for further analyses (Fig. 1). For a detailed description of these components, see Supplementary Note 1. Subsequent components in the temporal sorting were related to artifacts, such as head motion, physiological and scanner noise, cerebrospinal fluid, and white matter.

### Component modulation

**Fig. 2:**
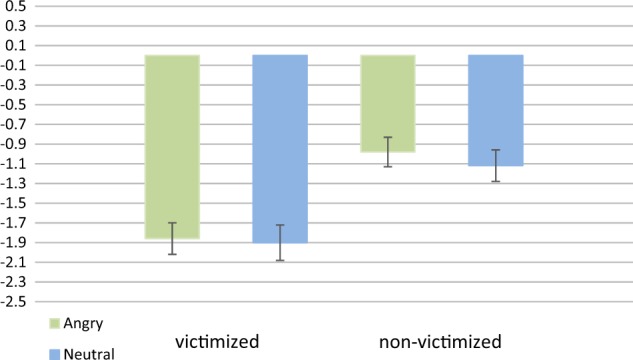
Results of the ICA in the sensorimotor component: representations of the beta weights per group and per task condition. Error bars represent standard errors

The component consisting of the (medial) sensorimotor network showed less activation in the victimized group compared to the nonvictimized group during the processing of angry faces (*F*(1,37) = 11.889, *p* = 0.000935). This effect was due to more deactivation of the sensorimotor network in the victimized group (Fig. 2). The component showed no main effect of task condition, or interaction effect between group and task condition. There were no significant differences observed for the other components.

## Discussion

This study investigated brain response during processing of angry facial expressions in a victimized and nonvictimized group of participants with a psychotic disorder. No differences were found between both groups in terms of regional brain activation and brain connectivity as analyzed with respectively GLM and generalized psychophysiological interaction (gPPI) analyses. Independent component analyses revealed more deactivation of the sensorimotor network in the victimized group compared to the nonvictimized group. This finding may be interpreted as a freeze reaction to threatening stimuli, previously seen in traumatized individuals.

We found activation in visual areas and key areas involved in processing of facial social information, in response to angry and neutral faces. These findings are in line with previous studies using an emotional faces paradigm in individuals with schizophrenia.^28–30^ While contrasting angry and neutral faces with baseline showed solid task activation, contrasting angry versus neutral faces however revealed no significant differences in brain activation at a corrected level. This could be explained by a similar pattern of brain response to both conditions (angry and neutral), which is consistent with previous studies. In these studies patients with schizophrenia responded to face- and word-stimuli, rated as neutral by healthy volunteers, as if they were emotional or arousing.^25,29^ Hall et al.^31^ systematically investigated brain response to neutral faces, fearful faces, and a baseline condition and revealed that patients with schizophrenia show an increased response of the amygdala to neutral faces. This explained findings of less amygdala activation to fearful faces compared with neutral faces, in a group of participants with schizophrenia compared to healthy individuals. Studies which contrast neural responses to emotional facial expressions versus responses to neutral expressions may underestimate the magnitude of brain activation as compared to studies which also contrast emotional facial expressions with a baseline condition consisting of for example scrambled faces.^29^

The lack of GLM and gPPI findings are in contrast with studies reporting differential brain activation to facial expressions between traumatized and nontraumatized groups.^19,20,32^ However, although victimization may be considered as a form of trauma, these studies included participants with subsequent PTSD symptoms which may have resulted in a more severely disabled sample in comparison to a nontraumatized control group. In addition, our group sizes were rather small, considering the heterogeneous nature of the sample with regard to illness duration and severity. Furthermore, the operationalization of victimization with an emphasis on overt aggressive behavior (threats, sexual, and other forms of physical violence) may have been not sensitive enough. Therefore, part of the participants now included in the control group might have been victim of more subtle forms of interpersonal violence, for example, by being bullied, social exclusion, rejection, or being mistreated as a result of stigmatization. Future studies are recommended to use more fine-grained measures of victimization.

In comparison to GLM and gPPI analyses, independent component analysis (ICA) analysis, which enables identification of networks in a data-driven manner, is more sensitive to detect subtle differences between participants.^33^ The ICA analysis revealed more deactivation of the sensorimotor network in the victimized group compared to the nonvictimized group. The sensorimotor network is implicated in processing and undertaking actions. Decreased activation in sensorimotor regions and decreased connectivity within the sensorimotor network has been previously associated with the common symptom “freezing of gait” in patients with Parkinson’s disease which refers to a brief, involuntary abortion of movement.^34,35^ Deactivation of the sensorimotor network in victimized patients may resemble to some extent the freeze response often observed in traumatized individuals.^36,37^ The freeze response is characterized by reduced body motion and bradycardia in response to threat.^38^ Roelofs et al.^37^ investigated freezing in a social threat context reporting bradycardia and reductions in body sway in response to angry faces compared to neutral and happy faces. It has been suggested that trauma affects motor responses, with increased trauma frequency being associated with more severe motor dysfunctions in conversion disorder.^39^ Hagenaars et al.^36^ have found that compared to people who had never experienced an aversive life event (e.g., sexual or physical assault and serious accidents), individuals who had experienced one aversive life event showed reduced body sway to unpleasant pictures. Moreover, people who had experienced multiple aversive life events showed reduced body sway in response to neutral, pleasant and unpleasant pictures indicating a cumulative effect of multiple trauma. Future studies may investigate whether this cumulative effect also applies to victimization incidents in people with a psychotic disorder. Similarly, it may be valuable to investigate whether the recency of an incident has an effect on the response to threatening information.

Although the prevalence rate of victimization is high for people with a psychotic disorder,^2^ and processing facial stimuli has been suggested to be associated with victimization in this group,^4^ this relationship has not been investigated explicitly. Moreover, no previous studies investigated factors associated with victimization in individuals with a psychotic disorder by means of functional magnetic resonance imaging (fMRI). The study also had several limitations. Limitations regarding the sample size and operationalization of victimization have been mentioned above. Two additional limitations regard the study design. First of all, the study was cross-sectional preventing causal inferences of the ICA results. Victimization may be traumatizing, leading to an exaggerated freeze response to threatening facial expressions previously reported in traumatized individuals. But the other way around, altered processing of facial expressions might be a risk factor of victimization in individuals with a psychotic disorder. As a next step, future longitudinal studies may give more insight in the direction of the effect. Second, the study lacks a healthy control group. Although there is clear evidence that individuals with a psychotic disorder have difficulties with facial expression recognition and processing,^6,7^ without a healthy control group, it is not possible to determine whether the differences in activation between the victimized and nonvictimized groups are specific to patients with psychosis.

The current study investigated brain response to angry faces in victimized and nonvictimized individuals with a psychotic disorder. The emotional faces task activated brain areas previously implicated in similar emotional faces paradigms. No differences in regional brain activation and connectivity between groups were found, which might be explained by the small sample size and presence of subtle forms of victimization in the nonvictimized group. The victimized group showed more deactivation of the sensorimotor network in response to angry faces compared to the non-victimized group. Although the study design does not allow for inferences regarding the direction of this effect, based on previous literature, this finding may be interpreted as a freeze reaction to threatening stimuli, previously seen in traumatized individuals. Our work builds up on previous studies investigating neural mechanisms of freezing in trauma that have reported alterations in certain brain areas,^40,41^ by adding the sensorimotor cortex as an important underlying brain region.

## Methods

### Participants

A total of 39 participants (26 male) were recruited as part of the “Beat victimization” study^42^ (Beatvic) which evaluates a body-oriented intervention that aims to prevent victimization of individuals with a psychotic disorder (Current Controlled Trials: ISRCTN21423535, van der Stouwe et al.^42^). We based our sample size on previous studies that have suggested that in principle, 20 participants are necessary per group to achieve sufficient power and acceptable reliability.^43,44^ The Beatvic study was approved by the local ethical committee (University Medical Center of Groningen, The Netherlands; METc protocol number: NL52202.042.15) and was performed in line with the declaration of Helsinki. Participants (age > 18) with a diagnosis in the psychotic spectrum according to DSM-IV-TR criteria were recruited from six mental health institutions in the Netherlands. Exclusion criteria were as follows: severe psychotic symptoms (PANSS mean positive symptoms > 5), substance dependence (not substance abuse), co-morbid neurological disorder, co-morbid personality disorder, estimated IQ < 70, pregnancy and, for participants in the MRI-study, extra MRI-compatibility related exclusion criteria such as metal implants and claustrophobia.

### Procedure

Onsite therapists selected potentially eligible patients based on in- and exclusion criteria and referred selected patients to the researchers. The research team subsequently contacted referred patients to provide information about the study and to ask whether they were interested in participating. Interested patients received an information letter and had a minimum of a 2-week period to consider participation. With patients who decided to participate, an intake was planned to verify in- and exclusion criteria by means of the miniSCAN and the PANSS interview and a written informed consent was obtained. Participants were asked whether they were interested to participate in the MRI substudy as well. If so, an MRI-checklist regarding MRI-compatibility related criteria was completed during the intake as well. Assessors were all trained in the miniSCAN and PANSS interview. In the current study, baseline MRI data were used.

### Measures

To assess sample characteristics miniSCAN interviews and PANSS interviews^45^ were performed and participants completed questions regarding age, gender, occupational status, living situation, illness characteristics, medication, and filled out the trauma screening questionnaire^46^ (TSQ). The TSQ is a ten-item instrument consisting of five re-experiencing and five arousal items, with a score of 6 as cut-off.

The victimization subscale of the Dutch Crime and Victimization Survey,^47^ a questionnaire that resembles the International Crime Victimization Survey was used to measure vicitmization. Because of our interest in the role of emotional face processing, we focussed on the items involving direct social interaction, i.e., the four items on personal victimization (see Table 3). The items were multiple choice questions consisting of four alternative answer options: “*Yes, last year*”, “*Yes, one year before*”, “*Yes, in the previous five years*”, and “*No*”. Participants that responded with “Yes” to any of these questions were considered victimized within the last 5 years. Literature on the validity of retrospective reports of victimization in people with a severe mental illness posit that retrospective assessments yield reliable information, and that false positives are rare.^48^

As part of the Beatvic study participants completed a gender discrimination task^49^ to assess implicit emotion processing and to keep participants’ attention to the stimuli. The task, included 16 blocks depicting photographs of individual angry, neutral, happy, and fearful faces acquired from the Karolinska Directed Emotional Faces database.^50^ Each block contained six trials, including three to five face trials from one emotion condition and one to three null trials consisting of a fixation cross. Face trials and null trials were mixed at random throughout each block. Each face trial consisted of a stimulus presented for 600 ms and an interstimulus interval of 200 ms during which a fixation cross was displayed. Participants were instructed to respond (indicate the gender) by means of a button box as fast as possible, before presentation of the following stimulus. Each null trial comprised presentation of a fixation cross for 600 ms, followed by an interstimulus interval of 200 ms.

### MRI acquisition

Neuroimaging data were acquired using a 3T Philips Intera MR-scanner (Best, the Netherlands), equipped with a SENSE 32-channel head coil. Functional images were acquired using a T2*-weighted echo-planar sequence and consisted of 39 descending axial slices, 3 mm thick, and no slice gap (repetition time = 2 s; echo time = 30 ms; flip angle = 90°; FOV = 192 × 192 × 117 mm, with an inplane matrix consisting of 64 × 61 voxels at 3 × 3 × 3 mm). All scans were oriented 10–20° to the AC-PC transverse plane to prevent artefacts from nasal cavities. The task consisted of 275 functional volumes. In addition, a high-resolution anatomical T1 image was recorded using the following parameter settings: voxel size 1 × 1 × 1 mm; 170 slices; TR = 9 ms; TE = 3.5 ms; slice thickness = 1 mm; 256 × 256 matrix; FOV 256 × 232 × 170 mm.

### Data analysis

Demographic and clinical differences between the treatment and control group were tested using a Pearson chi-squared test for categorical variables or Fisher’s exact tests in case expected cell counts <5. Continuous variables were tested with independent *t* tests and in case these variables were not normally distributed by means of Mann–Whitney *U* tests.

Reaction times (RT) and accuracies (Acc) on the gender discrimination task were analyzed by a condition (angry, neutral) × group (victimized and nonvictimized) repeated measures ANOVA, with group defined as a between-subjects variable and condition as a within-subjects variable.

Neuroimaging data were preprocessed using Statistical Parametric Mapping 12 version 6470 (Welcome Department of Cognitive Neurology, UCL) in Matlab version 7.8.0 (Mathworks, Natick USA). Functional images were realigned and then co-registered to the anatomical T1 image. Next, the images were normalized to Montreal Neurological Institute (MNI) space using the MNI152 template and smoothed using an 8 mm full width at half maximum Gaussian kernel. Due to excessive movement according to realignment (>3 mm), data of one participant were excluded from further analysis. In case of excessive movement (>3 mm) at the end of the task, we excluded the last part of the volumes. This was the case for five participants and resulted in exclusion of 1.4% of all acquired volumes.

For first-level analyses, general linear models (GLM), including four task regressors (angry, neutral, happy, and fearful), defined as onset times per trial, were convolved with the canonical hemodynamic response function (HRF). To correct for motion, six motion parameters and their first derivatives were added. In addition, framewise displacement (FD) was calculated and included as a regressor. Motion was deemed excessive when FD > 0.9 for a certain volume.^51^ This was the case in 12 participants and resulted in exclusion of 0.5% of the acquired volumes. Because we were particularly interested in brain response to angry facial expressions in the context of victimization, for each participant, the following contrasts were computed: (1) angry > baseline, (2) neutral > baseline, and (3) angry > neutral.

Single-subject contrast images of the contrasts angry > baseline, neutral > baseline and angry > neutral were used to perform three one-sample *t* tests at second level to examine main task effects. A two-sample *t* test on the angry > neutral contrast images was performed to compare the victimized and the nonvictimized group. Medication use was entered as covariate of no interest in all analyses by means of a dummy variable (antipsychotic medication yes/no; antidepressant medication yes/no). Previous mentioned key areas involved in processing facial social information, the amygdala, STS, FG, insula, orbitofrontal cortex, ACC, and the cuneus were defined as ROI). A composite mask including a total of 39527 voxels (1 × 1 × 1) was constructed by means of the WFU pickatlas.^52^ To correct for multiple comparisons, contrast images were thresholded at *p* < 0.05 FWE cluster-level for the extent of the ROI-mask with an initial threshold of *p* < 0.001 uncorrected.

gPPI analyses were used to investigate the functional connectivity between a seed region (e.g., the amygdala) and the rest of the brain in response to the task conditions.^53^ To construct the seed region we used the WFU pickatlas.

At first-level, for each participant a separate gPPI model was estimated for the seed region. To obtain the physiological variable, BOLD signals were extracted from this region. Hemodynamic deconvolution was performed on the extracted time series to remove the effects of the canonical HRF. The resulting time series were multiplied by the task regressors (angry, neutral, happy, and fearful) and convolved with the HRF, eventually resulting in nine regressors: four task conditions, one for the time series of the seed and four seed × condition interaction regressors. To identify regions that showed stronger functional connectivity with the seed during presentation of an angry compared to a neutral facial expression, the contrast angry > neutral was created by subtracting the gPPI interaction regressor of the neutral condition from the interaction regressor of the angry condition.

On second level, the contrast images were entered into a one-sample *t* test to examine task connectivity in the whole sample. A two-sample *t* test was performed to compare the victimized and nonvictimized group. We applied a threshold of *p* < 0.05 FWE at cluster-level for the extent of the ROI-mask with an initial threshold of *p* < 0.001 uncorrected.

ICA was performed with the Group ICA of fMRI Toolbox (GIFT; version 3.0b; MATLAB Software), which was implemented in Matlab version 2013b.^54^ The number of independent components was estimated using maximum description length and Akaike’s criteria, which resulted in 30 components. For all participants, images were decomposed into 30 spatially independent components using the Infomax algorithm. Single-subject time courses and spatial maps were back-reconstructed by means of spatial-temporal regression. Subsequently, a group ICA was performed and its stability was assessed by performing an ICASSO on 20 iterations.^55^

To calculate the association between the time courses of the independent components with the conditions of the emotional faces task, design matrices derived from the GLM analysis were entered in the temporal sorting function (multiple regression) in GIFT to calculate the correlation between the components and the task. The resulting beta weights were entered into a two-way ANOVA with two groups (victimized and nonvictimized) and two conditions (angry and neutral faces) to test for group differences in component (de)activation. Following Bonferroni, *p* values were divided by the number of components that were investigated to correct for multiple testing.

### Reporting summary

Further information on research design is available in the Nature Research Reporting Summary linked to this article.

## Supplementary information

Supplementary Note 1.

Reporting summary

## Data Availability

The data that support the findings of this study are available from the corresponding author upon reasonable request.

## Supplementary information

**Supplementary information** accompanies the paper on the *npj Schizophrenia* website (10.1038/s41537-019-0082-z).
