# Supplementary material for: Neural correlates of victimization in psychosis: differences in brain response to angry faces
Source: NPJ Schizophr. 2019 Sep 9;5:14. doi: 10.1038/s41537-019-0082-z (PMC6733807; doi:10.1038/s41537-019-0082-z)
Supplement: Supplementary file 1 — Supplementary Note 1. [file 41537_2019_82_MOESM1_ESM.pdf]

Supplementary Note 1. Detailed description of components

Component A (salience network, r=0.14) consisted of a bilateral network including the insula, superior frontal regions and the anterior cingulate cortex. Component B (fronto-parietal network, r=0.13) comprised the dorsal anterior cingulate cortex extending to the motor cortex, the superior frontal gyrus, the middle frontal gyrus and the bilateral insula. Component C (anterior default mode network (DMN), r=0.09) revealed a pattern of medial superior frontal and middle frontal regions, the medial orbitofrontal gyrus, the anterior and posterior cingulate cortex and the thalamus. Component D (posterior default mode network, r=0.10) included the posterior and middle cingulate gyrus, the precuneus and the cuneus, the angular gyrus, the middle occipital gyrus, the medial orbitofrontal cortex, the superior temporal gyrus and the thalamus. Component E (sensorimotor medial, r=0.11) contained the precentral and postcentral gyrus, the supplementary motor area, the supramarginal gyrus and the inferior and superior parietal lobule. Component F (sensorimotor lateral, r=0.20) included the postcentral gyrus, the paracentral lobule, the precuneus and the supplementary motor area. Component G (visual network, r=0.11) comprised middle occipital and superior occipital regions, the cuneus, the calcarine gyrus and the lingual gyrus.

Component A (visual network, r=x) comprised inferior, middle and superior occipital regions, the cuneus, the calcarine gyrus and the lingual. Component B (dorsal attention network, r=x) postcentral, precentral, superior parietal, inferior parietal, supplementary motor area, middle cingulate gyrus, frontal medial orbital gyrus. Component C (dorsal attention network, r=x) inferior frontal gyrus, fusiform, middle temporal gyrus, middle occipital gyrus. Component D (cerebellum, r=x) .. Component E (anterior default mode network, r=x)
